# A Case–Control Study Examining the Differences in Vitamin D Levels Between Individuals With Rosacea and Healthy Individuals

**DOI:** 10.1111/jocd.70141

**Published:** 2025-06-10

**Authors:** Gita Faghihi, Fatemeh Mohaghegh, Mohammad Shoushtarizadeh, Nazila Poostiyan, Sayed Mohsen Hosseini, Arian Khosravi

**Affiliations:** ^1^ Skin Diseases and Leishmaniasis Research Center, Department of Dermatology Isfahan University of Medical Sciences Isfahan Iran

**Keywords:** risk factor, rosacea, vitamin D

## Abstract

**Background:**

Rosacea is an inflammatory and chronic skin condition that may be affected by many factors. The purpose of this study is to compare the level of vitamin D in two groups of cases (suffering from rosacea) and controls (healthy).

**Methods:**

This case–control study was conducted in 2022 on 62 patients referred to Sedigheh Tahereh dermatology clinic, Isfahan, Iran. The criteria for entering the case group are to have rosacea based on the updated phenotype‐based diagnosis and classification system. Demographic variables such as age and gender as well as vitamin D levels were recorded for both case and control groups.

**Results:**

The mean of vitamin D level among patients with rosacea (29.9 ± 2.95) was higher than the control group (24.5 ± 2.14), but the difference was not statistically significant (*p* > 0.05). There was no significant difference in the mean of vitamin D level between rosacea patients and controls in gender or severity subgroups.

**Conclusion:**

There is no significant difference in vitamin D levels between patients with rosacea and healthy individuals.

## 1 Introduction

Rosacea is a persistent inflammatory skin disorder characterized by the presence of papules and pustules, facial redness, episodes of flushing, and the appearance of telangiectasia [1]. Patients may experience secondary symptoms, including skin burning, tingling, dryness, edema, eye involvement, and phymatous changes [2]. This chronic condition significantly impacts patients' quality of life, causing psychological distress, social anxiety, and decreased self‐esteem due to its visible manifestations on facial skin [2]. According to estimates, rosacea affects about 5.46% of the world's population [3]. Notably, its prevalence reaches up to 22% in some races [2]. Rosacea has four subtypes: erythematous‐telangiectatic, papulo‐pustular, phymatous, and ocular. The erythematous‐telangiectatic type is the most prevalent form [4]. Despite its considerable prevalence and impact, the exact pathophysiological mechanisms underlying rosacea remain incompletely understood. Current evidence suggests a complex interplay of genetic predisposition, vascular abnormalities, inflammatory pathways, and dysregulation of the innate immune system [5]. Microorganisms, UV rays, nutrition, temperature changes, skin barrier disruption, stress, and hormones can all heighten the innate immune response, worsening rosacea symptoms [5].

Vitamin D, a fat‐soluble vitamin, significantly influences the innate and adaptive immune systems by regulating antimicrobial peptides, T‐cell activation, and dendritic cell maturation. This crucial vitamin modulates inflammation, keratinocyte proliferation, and differentiation and plays a vital role in maintaining skin barrier integrity—all processes potentially relevant to rosacea pathogenesis [6]. It is synthesized in the skin when UV rays from sunlight trigger its production. The vitamin from sunlight, food, and supplements is inactive and requires two hydroxylations in the liver and kidney to activate [6]. Recent interest in the relationship between vitamin D and inflammatory skin disorders has led to investigations of its potential role in rosacea, though with conflicting results. According to specific research, rosacea sufferers had lower vitamin D serum levels than healthy controls [7]. However, other researchers contend that people with rosacea have higher vitamin D levels [8, 9]. These contradictory findings may stem from methodological differences, regional variations in sun exposure, seasonal fluctuations in vitamin D levels, or heterogeneity in rosacea subtypes among study populations. The link between vitamin D levels and rosacea remains unclear due to inconsistent findings. Elucidating this relationship could provide valuable insights into rosacea pathophysiology and potentially inform novel therapeutic approaches. This study compares vitamin D levels in rosacea patients with healthy controls.

## 2 Methodology

### 2.1 Study Design and Setting

This cross‐sectional study was conducted at the dermatology clinic of Isfahan University of Medical Sciences in 2022. The study base consisted of individuals attending the dermatology clinic for routine check‐ups or dermatological concerns.

### 2.2 Participants

The study included 62 participants: 31 with rosacea (case group) and 31 healthy individuals (control group).

### 2.3 Inclusion and Exclusion Criteria

- Case group (rosacea patients):
  ○ *Inclusion criteria*:
    1. Individuals diagnosed with rosacea based on revised diagnostic criteria, including:
      - Persistent centrofacial erythema with episodes of exacerbation.
      - The presence of phymatous alterations [9].
    2. Age between 18 and 60 years.
  ○ *Exclusion criteria*:
    1. Presence of osteoporosis, connective tissue diseases, or organ failure.
    2. Pregnancy or breastfeeding.
    3. Use of treatments affecting vitamin D levels (e.g., oral calcium or vitamin D supplements) within the past 3 months.
- Control group (healthy individuals):
  ○ *Inclusion criteria*:
    1. Healthy individuals with no history or clinical signs of rosacea.
    2. Age between 18 and 60 years.
  ○ *Exclusion criteria*:
    1. Presence of any chronic dermatological or systemic diseases.
    2. Pregnancy or breastfeeding.
    3. Use of treatments affecting vitamin D levels (e.g., oral calcium or vitamin D supplements) within the past 3 months.

### 2.4 Disease Severity Classification

Disease severity in the rosacea group was classified as mild, moderate, or severe based on the National Rosacea Society (NRS) grading system, which evaluates the extent of erythema, papules/pustules, and phymatous changes.

### 2.5 Data Collection

Demographic data (age, sex, and disease severity) and vitamin D levels were collected for both groups. Vitamin D levels were measured using enzyme‐linked immunosorbent assay (ELISA).

### 2.6 Statistical Analysis

The normality of continuous variables was assessed using the Shapiro–Wilk test (*p* > 0.05). Continuous variables were expressed as mean ± standard deviation (SD), while categorical variables were presented as count (percentage). Pearson's chi‐square or Fisher's exact test was used for group comparisons for categorical variables. An independent *t*‐test was applied to compare vitamin D levels between groups. The relationship between vitamin D and rosacea was assessed using logistic regression, adjusted for potential confounders such as age. Statistical significance was set at *p* < 0.05. All analyses were performed using SPSS version 20.0.

### 2.7 Ethical Considerations

This study adhered to the tenets of the Declaration of Helsinki. The ethics committee of Isfahan University of Medical Sciences approved the study with approval ID IR.MUI.MED.REC.1401.034. Written informed consent was obtained from all participants before their inclusion in the study.

### 2.8 Sample Size Calculation

The sample size for this study was calculated based on the following formula for comparing two independent means [9]:

$$n=\frac{{\left(Z1-\alpha /2+Z1-\beta \right)}^{2}*\left(S1^{2}+S2^{2}\right)}{d^{2}}$$

where:

- *α* = 0.05 (95% confidence level) → Z1 − α/2 = 1.96.
- *β* = 0.20 (80% power) → Z1 − *β* = 0.84.
- S1 = 9.9 (standard deviation of group 1).
- S2 = 7.9 (standard deviation of group 2).
- *d* = 6.4 (effect size or clinically meaningful difference).

Substituting the values into the formula:

$$n=\frac{1257.6}{40.96}\approx 31$$

Thus, 31 participants per group were required to achieve adequate statistical power for this study.

## 3 Results

The mean age of rosacea patients was 37.3 ± 9.8 years (17–58 years). The healthy group's mean age was 39.8 ± 9.4 years (range: 19–56 years). No significant difference was observed between the two groups (*p* = 0.373) (Table 1).

**TABLE 1:** Demographic characteristics data according to the study groups.

Characteristics	Group		*p*
	Rosacea (*n* = 31)	Healthy (*n* = 31)	
Age year (±SD)	37.7 (±9.8)	39.8 (±9.4)	0.373
Gender *N* (%)			
Female	23 (50%)	23 (50%)	> 0.999^a^
Male	8 (50%)	8 (50%)	

*Note:* Data are shown as mean (SD) for continuous variables and frequency (%) for categorical variables.

^a^ *p*‐value was obtained from the Chi‐Square test.

Gender distribution did not show a significant difference between the groups (*p* > 0.999) (Table 1). Detailed demographics based on disease status are provided in Table 1. In the rosacea group, 14 patients (45.2%) had mild disease, eight (25.8%) had moderate disease, and nine (29%) had severe disease (Table 2).

**TABLE 2:** Comparison of vitamin D levels between the healthy group and the rosacea patients with different severity groups.

Rosacea Severity	*N*	Mean	95% Confidence Interval for Mean		*p*
			Lower bound	Upper bound	
Healthy group	31	24.52	19.99	29.06	0.269
Mild	14	26.32	15.86	36.78	
Moderate	8	29.69	21.68	37.69	
Severe	9	35.56	19.21	51.91	

*Note:* Data are shown as mean with 95% CI, and *p*‐values result from one‐way ANOVA.

The mean vitamin D level in rosacea patients was 29.9 ± 2.95, compared to 24.5 ± 2.14 in the healthy group. However, this difference was not statistically significant (*p* > 0.05). Similarly, no significant difference in vitamin D status was found between the two groups (*p* > 0.05) (Table 3).

**TABLE 3:** Comparison of vitamin D levels between the study groups.

Characteristics	Group		Mean difference 95% CI	*p*
	Rosacea (*n* = 31)	Healthy (*n* = 31)		
25 (OH) D (ng/mL)	29.9 (24.6–36.3)	24.5 (20.6–28.7)	5.4 (−2.18–13.0)	0.159
Vitamin D level *N* (%)				
Deficiency	11 (44%)	14 (56%)	0.234^a^	
Insufficiency	5 (35.7%)	9 (64.3%)		
Optimal level	12 (66.7%)	6 (33.1%)		
High level	3 (75%)	1 (25%)		

*Note:* Data are shown as mean (95% CI) for continuous variables and frequency (%) for categorical variables.

^a^ *p*‐value was obtained from the Fisher exact test.

Logistic regression, adjusted for age, also showed no significant association between vitamin D levels and rosacea (OR = 1.04, 95% CI: 0.995 to 1.10) (Table 1).

**TABLE 4:** Comparison of vitamin D levels between rosacea and healthy groups according to gender.

Gender	Rosacea	Healthy	*p*
Female	30.38 (23.27–38.70)	24.05 (19.45–29.96)	0.202
Male	28.40 (21.28–34.56)	25.89 (18.98–33.12)	0.629

*Note:* Data are shown as mean (95% CI), and *p*‐values are the results from the independent *t*‐test.

There was no significant difference in vitamin D levels between rosacea patients and controls of both genders (*p* > 0.05). Vitamin D levels did not significantly differ based on rosacea severity (*p* = 0.269) (Table 4).

## 4 Discussion

Vitamin D levels in rosacea patients have been debated. Our cross‐sectional study found no significant differences in vitamin D levels between rosacea patients and healthy controls, regardless of disease severity or gender.

Several studies have explored vitamin D levels in rosacea, revealing ongoing contradictory findings. In 2013, Ekiz et al. [9] first reported that vitamin D levels in rosacea patients were significantly higher than in controls, suggesting a possible positive role in disease development. Supporting this, case–control studies by Gürel et al. [4] and Akdogan et al. [8] in 2018, involving 50 and 60 patients, respectively, corroborated these results.

In 2018, Park et al. [7] questioned vitamin D's positive role in rosacea, noting lower levels in patients than controls, suggesting further investigation. They also found a significant link between higher cathelicidin expression and rosacea. In 2021, Hagag et al.'s [6] study on vitamin D levels in 30 rosacea patients and 20 matched controls reignited debates on the topic.

In 2023, Mao et al. [10] analyzed 25‐hydroxyvitamin D levels in nearly 370 000 individuals, including about 2000 with rosacea, finding an inverse relationship between serum vitamin D concentration and rosacea development. They suggested that vitamin D may have a protective role and emphasized the need for further research on vitamin D supplementation as prevention.

However, a study of 31 patients in the case and control groups did not support the idea that serum vitamin D levels significantly affect rosacea development. Current inconsistent results may be influenced by factors like age, sex, race, and the severity of vitamin D deficiency [11, 12], along with unstudied genetic and environmental factors such as sun exposure.

More case–control and cohort studies with larger populations, systematic reviews, and meta‐analyses can clarify this issue. Long‐term randomized clinical trials on vitamin D supplementation's effect on rosacea would also help.

This study had several limitations. First, the small sample size reduces the statistical power and generalizability of the results. Second, the vitamin D level in individuals is influenced by several factors, such as body mass index, seasonal variations, and dietary habits. However, these confounding factors were not controlled or adjusted for in the study, which could affect the interpretation of the results. Additionally, the cross‐sectional design without patient follow‐up limits the ability to establish causal relationships and may introduce bias. Future studies with larger sample sizes, longitudinal designs, and adjustments for potential confounders are recommended to address these limitations.

Also, further work needs to be done to determine whether the results of this study differ from other studies in terms of differences in populations, methods, or diagnostic criteria.

## 5 Conclusion

In conclusion, this study found no significant association between vitamin D levels and rosacea, even after age and sex adjustment. However, it is vital to acknowledge the limitations of this study, particularly the relatively small sample size. A larger sample size might provide more robust statistical power to detect potential group associations or differences. Future studies with a more extensive and diverse population are recommended to explore the relationship between vitamin D and rosacea further.

## Author Contributions

Gita Faghihi and Nazila Poostiyan contributed to designing the study, patients care and data collection and interpretation; Arian Khosravi and Fatemeh Mohaghegh contributed to the data collection, literature search and drafted the manuscript; Sayed Mohsen Hosseini contributed to data analysis. Gita Faghihi, Fatemeh Mohaghegh and Nazila Poostiyan reviewed and finalized the manuscript. All authors read and approved the final manuscript.

## Ethics Statement

This study adhered to the tenets of the Declaration of Helsinki. The ethics committee of Isfahan University of medical sciences approved the study. Written informed consent to participate was obtained from all patients.

## Consent

The authors express their gratitude to the patients for allowing the publication.

## Conflicts of Interest

The authors declare no conflicts of interest.

## Data Availability

This case–control study was conducted in 2022 on 62 patients referred to Sedigheh Tahereh dermatology clinic, Isfahan, Iran. The authors confirm that the data supporting the findings of this study are available within the article and its Supporting Information.
